# Comparative assessment of DeepSeek-R1 and GPT-4 for structured ultrasound reporting of adnexal masses

**DOI:** 10.3389/fonc.2026.1904488

**Published:** 2026-08-11

**Authors:** Mengjuan Zhang, Bin Wang, Ran Li, Xinwu Cui, Xiaofeng Zhang, Jingzhe Wang, Lan Jia, Lijuan Guo, Yan Kong

**Affiliations:** 1 Department of Medical Ultrasound, The First Affiliated Hospital of Xinxiang Medical University, Xinxiang, Henan, China; 2 Department of Respiratory Intensive Care, The First Affiliated Hospital of Xinxiang Medical University, Xinxiang, Henan, China; 3 Department of Medical Ultrasound, Tongji Hospital, Tongji Medical College and State Key Laboratory for Diagnosis and Treatment of Severe Zoonotic Infectious Diseases, Huazhong University of Science and Technology, Wuhan, Hubei, China

**Keywords:** adnexal masses, DeepSeek-R1, GPT-4, structured reports, ultrasound diagnosis

## Abstract

**Objective:**

This study primarily evaluated the ability of two large language models (DeepSeek-R1 and GPT-4) to generate structured ultrasound reports from free-text adnexal mass reports. Secondarily, we assessed their accuracy in O-RADS classification and management recommendations, with an exploratory analysis of their performance in benign versus malignant discrimination.

**Methods:**

This study included 215 free-text ultrasound reports of adnexal masses (AMs) between July 2024 and March 2025. Each report was processed three times per model; majority voting was used to determine the final output. Three senior radiologists, blinded to model identity, evaluated structured reports against a predefined template, O-RADS categories, and management guidelines. Histopathology served as the reference standard for benign/malignant discrimination; while expert consensus served as the reference for the other endpoints.

**Results:**

GPT-4 showed numerically better performance than DeepSeek-R1 in generating structured reports (99.5% vs. 96.7%, p = 0.07).DeepSeek-R1 outperformed GPT-4 in O-RADS accuracy (64.4% vs. 52.9%, p < 0.001) and appropriate management recommendations (66.4% vs. 58.0%, p = 0.007). Both models demonstrated good discrimination ability for benign versus malignant classification (AUC > 0.80 for both).

**Conclusion:**

Both DeepSeek-R1 and GPT-4 demonstrate potential for automating structured ultrasound reporting of AMs, with DeepSeek-R1 showing superior performance in classification and recommendations. However, their diagnostic performance in discriminating benign from malignant masses, while promising, requires further refinement before independent clinical application. These findings support their role as assistive tools, pending integration with image data and prospective validation.

## 1 Introduction

In recent years, artificial intelligence (AI) has progressively integrated into various aspects of our lives, impacting numerous industries to varying degrees. The field of healthcare is no exception. Advances in AI have driven numerous innovations in medicine, particularly through large language models (LLMs), which exhibit potential in multimodal data processing and knowledge reasoning, including applications in medical imaging (1–5).

Representative LLMs such as GPT-4, leveraging natural language processing and multimodal fusion capabilities, demonstrate potential applications in assisting the generation of structured radiology reports and integrating patient clinical data with imaging features (1, 6, 7). More recently, the emergence of DeepSeek series models has shown competitive performance in several natural language processing tasks (8–10). However, to date, publicly available studies specifically evaluating DeepSeek for assisted generation of structured imaging reports or analysis of medical images remain extremely limited (11). Thus, its potential and quality in aiding structured report generation thus warrant further investigation and validation.

Recent studies have extended the application of LLMs and AI models to RADS-based reporting systems and ovarian tumor classification (12–14). Adnexal masses (AMs) represent a common condition in women. Malignant tumors in the adnexal region are the second most common cancers after breast cancer and constitute a leading cause of death from gynecologic malignancies (15, 16). Consequently, accurately distinguishing between benign and malignant AMs is crucial for individualized patient management (17, 18). Ultrasound is the most frequently used modality for evaluating AMs. However, variability in ultrasound reports can limit the effectiveness of ultrasound assessment and impact clinical management (19, 20). To address this, the American College of Radiology published the Ovarian-Adnexal Reporting and Data System (O-RADS). This system utilizes standardized lexicons, encompasses all risk categories, and provides corresponding management recommendations for each, aiming to enhance diagnostic accuracy and reproducibility (21, 22). Nevertheless, as radiologists must simultaneously perform image interpretation, match standardized lexicon terms, and assign risk categories during the scanning process, developing efficient methods for generating structured ultrasound reports for AMs becomes particularly important. However, to our knowledge, no studies have yet evaluated the performance of DeepSeek-1 and GPT-4 in generating structured reports for AMs.

To address this gap, this study aims to compare DeepSeek-R1 and GPT-4 across four distinct tasks: (1) generating structured ultrasound reports from free-text narratives (primary outcome); (2) assigning O-RADS categories (secondary outcome); (3) providing management recommendations (secondary outcome); and (4) exploring their performance in benign versus malignant discrimination (exploratory analysis).

## 2 Methods

### 2.1 Study design and ethical approval

This retrospective study was approved by the institutional review board of the participating hospital. Informed consent was exempted due to the retrospective nature of the study. No personally identifiable information was provided to DeepSeek-R1 and GPT-4. All patient data were anonymized prior to analysis.

### 2.2 Data collection and case selection

We collected 215 ultrasound reports of AMs from patients who underwent surgery for AMs and obtained postoperative pathological results at our hospital between July 2024 and March 2025, encompassing a total of 295 masses (184 benign and 111 malignant). The original ultrasound reports were written in Chinese and were directly input into the models in their original language without translation. Both DeepSeek-R1 and GPT-4 support Chinese language processing, and the prompts were also presented in Chinese to maintain consistency with the clinical context and avoid potential information loss during translation. The inclusion criteria were: patients aged ≥18 years who underwent transvaginal ultrasound examination and had a formal ultrasound report issued. The exclusion criteria were: (i) patients with a history of prior surgical resection of AMs or radiotherapy/chemotherapy; (ii) patients with indeterminate pathological results;(iii) non-adnexal masses or complex lesions; (iv) ultrasound reports that were extremely brief or lacked essential information.

### 2.3 Model specifications and access settings

Two large language models were evaluated: DeepSeek-R1 and GPT-4.

DeepSeek-R1: version R1-202505 (DeepSeek Inc., Beijing, China). Accessed via the official API from June 1 to June 10, 2025. Parameter settings: temperature = 0.7, top_p = 0.9, max_tokens = 2048.

GPT-4: version GPT-4-2024-04-09 (OpenAI Inc., San Francisco, CA, USA). The model was accessed via the OpenAI API during the same period (June 1–10, 2025). Parameter settings were identical to those of DeepSeek-R1 (temperature = 0.7, top_p = 0.9, max_tokens = 2048). No system prompt was used; only the user prompt described below was provided. For each case, a new conversation session was started to avoid context carry-over between reports.

### 2.4 Prompting strategy and output generation

**Figure 1:**
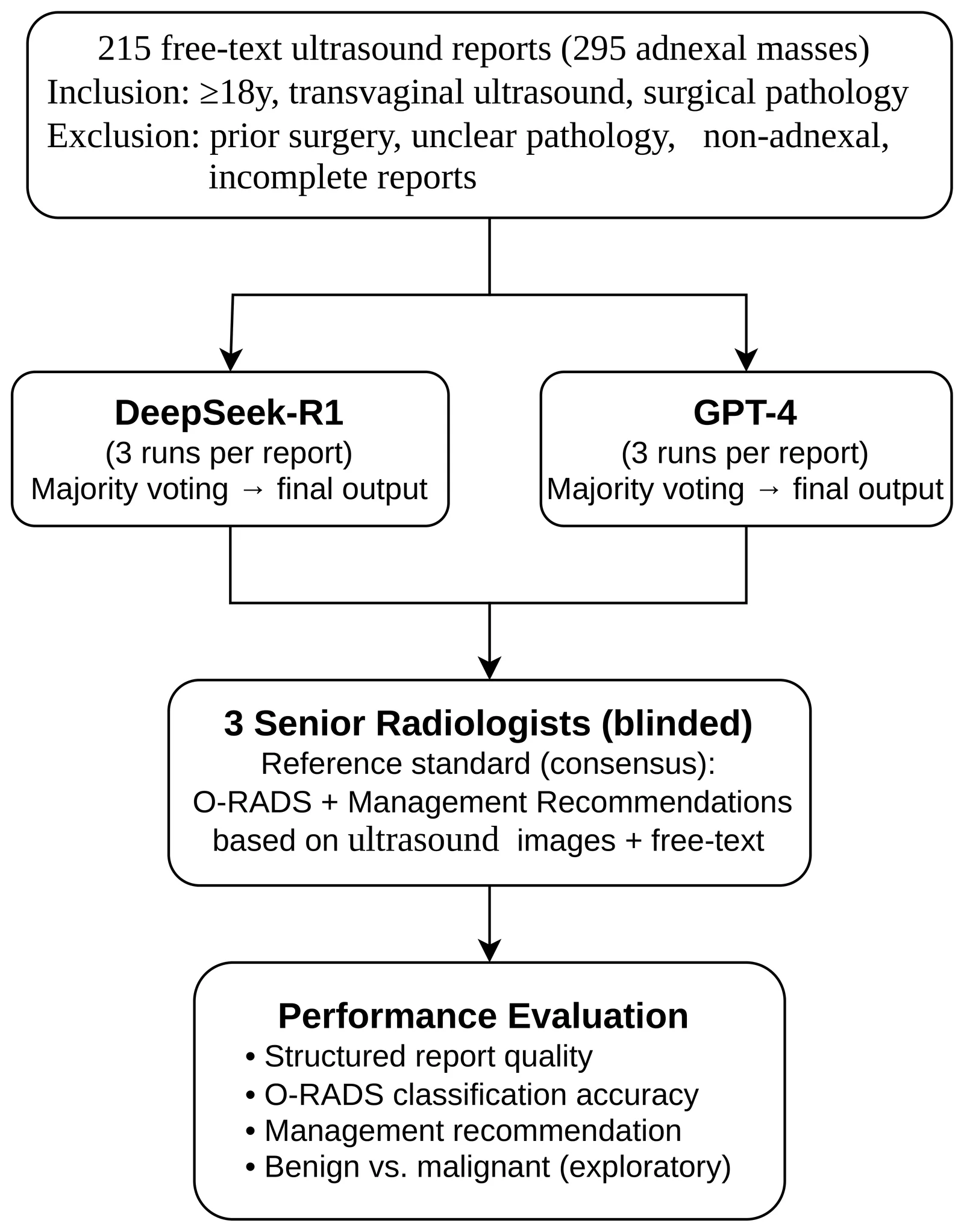
Flowchart for processing free-text adnexal mass ultrasound reports and output evaluation using DeepSeek-R1 and GPT-4.

The workflow of this study is illustrated in **Figure 1**. The complete prompt template, including the user prompt and all parameter settings, is provided in **Supplementary Material S2**. To optimize the output for the desired content, we crafted a detailed prompt: *“Please generate a structured medical ultrasound report based on the following free-text description of an AM. The report should include patient information, ultrasound findings, O-RADS categories, and management recommendations based on the O-RADS guidelines (2022 version).”* For patients with multiple AMs, all masses were included in the same free-text report, and the models were instructed to identify each mass separately and generate individual structured report sections with corresponding O-RADS categories and management recommendations for each lesion. Each free-text report was independently input into DeepSeek-R1 and GPT-4 three times. For each model–report pair, majority voting was used to determine the final output (i.e., the result that appeared at least twice among the three runs; if all three outputs differed, the first run was used as the tie-breaker). The stability of the three repeated runs was assessed separately as a secondary analysis and is reported in **Supplementary Table 1**. An example of a free‑text ultrasound report and the corresponding structured report generated by DeepSeek‑R1 is provided in **Supplementary Material S3**.

### 2.5 Reference standards

Different reference standards were used for different endpoints:

#### 2.5.1 Structured report generation

The reference standard was a predefined structured report template (provided in **Supplementary Material S1**). This template was developed based on routine clinical reporting standards and the ACR O−RADS guidelines. It required the following elements to be explicitly extracted or derived from the original free−text report: patient information (age, menopausal status), clinical history (presenting symptoms, relevant history), global ultrasound description of the uterus and contralateral adnexa, and for each adnexal mass: location (right/left), size (three dimensions), shape and margins, internal echogenicity, posterior acoustic features, color Doppler flow findings, O−RADS category, and a management recommendation consistent with O−RADS guidelines.

#### 2.5.2 O-RADS classification and management recommendations

The reference standard was the consensus judgment of three senior radiologists (with >20, 15, and 10 years of experience in gynecological ultrasound, respectively). They independently assigned O−RADS categories based on original ultrasound images and free−text reports, and evaluated the appropriateness of management recommendations according to the O−RADS guidelines (2022 version). Disagreements were resolved by discussion. Notably, the radiologists had access to both original ultrasound images and free−text reports, whereas the LLMs processed only the narrative reports. This information imbalance is acknowledged and considered when interpreting the results. Histopathology was not used as the reference standard for O−RADS classification or management recommendations.

#### 2.5.3 Benign vs. malignant discrimination

The reference standard was surgical histopathology (benign vs. malignant).

### 2.6 Evaluation of model outputs

#### 2.6.1 Structured report assessment

A three-point scale was used to evaluate the quality of the generated structured reports:(i) Structured–containing all prompt-required information without errors; (ii) Partially structured–including most required information while remaining error-free; (iii) Unstructured or erroneous–demonstrating either structural failure or clinically inaccurate content.

#### 2.6.2 O-RADS classification accuracy

Model−assigned O−RADS categories were compared against the expert consensus categories and classified as: (i) Correct: Model category matched the expert consensus; (ii) Incorrect: Model category differed from the expert consensus; (iii) Unclassifiable: Model did not output an O−RADS category or the output was uninterpretable. Unclassifiable outputs were counted as incorrect in the accuracy calculation (**Table 1**) to reflect the clinical scenario where an uninterpretable output is as non−actionable as an incorrect one.

**Table 1:** Distribution of the scale for structured reports, O-RADS categories, management recommendations, and predictive ability of DeepSeek-R1 and GPT-4.

Outcome	DeepSeek-R1	GPT-4	*P* value*	OR	95% CI
Structured reports (n=215)					
Structured	96.7%(208/215)	99.5%(214/215)			
Structured but incomplete	0%(0/215)	0%(0/215)			
Unstructured or contains incorrect information	3.3%(7/215)	0.5%(1/215)			
**Structured (Primary Outcome)**	96.7%(208/215)	99.5%(214/215)	0.070	0.14	(0.018–1.160)
O-RADS categories (n=295)					
Correct	64.4%(190/295)	52.9%(156/295)			
Incorrect	34.9%(103/295)	43.1%(127/295)			
Not categorized	0.7%(2/295)	4.1%(12/295)			
**Correct (Secondary Outcome)**	64.4%(190/295)	52.9%(156/295)	<0.001	2.42	(1.50–3.89)
Management recommendations (n=295)					
Reasonable	66.4%(196/295)	58.0%(171/295)			
Partially reasonable	9.8%(29/295)	9.1%(27/295)			
Unreasonable or not provided	23.7%(70/295)	32.9%(97/295)			
**Reasonable (Secondary Outcome)**	66.4%(196/295)	58.0%(171/295)	0.007	1.89	(1.20–2.99)

*For the primary outcome, P < 0.05 was considered significant (McNemar’s test). For secondary outcomes, the Benjamini-Hochberg correction was applied (threshold: P < 0.017). “Partially structured” and “Unstructured” were pooled as non-successful for the primary analysis. Bold values indicate the primary and secondary endpoint comparisons between the two models: the primary outcome (structured report generation) and secondary outcomes (O‑RADS classification accuracy and management recommendation appropriateness). P values for secondary outcomes were adjusted using the Benjamini‑Hochberg correction (significance threshold P < 0.017).

#### 2.6.3 Appropriateness of management recommendations

Based on the O-RADS guidelines (2022 version), model-generated management recommendations were classified as:(i) Appropriate (strictly adhering to O-RADS guideline recommendations), (ii) Partially appropriate (generally consistent with O-RADS recommendations but potentially containing incomplete or redundant elements), (iii) Inappropriate or not provided (deviating from O-RADS recommendations or absent).

### 2.7 Diagnostic performance for benign vs. malignant discrimination

Using histopathology as the gold standard, we evaluated the predictive performance of the models based on their assigned O-RADS categories. O-RADS 1–3 were defined as“predicted benign”and O-RADS 4–5 as“predicted malignant”. For lesion-specific endpoints (structured report generation and O-RADS classification), each mass was treated as an independent unit of analysis (n = 295), regardless of whether multiple masses originated from the same patient. For patient-level benign/malignant discrimination, a patient was classified as malignant if any mass was histopathologically confirmed as malignant (i.e., the highest-risk lesion determined the patient-level classification). Sensitivity, specificity, positive predictive value, negative predictive value, accuracy, and area under the receiver operating characteristic curve (AUC) were calculated. The diagnostic performance of senior radiologists was based on their consensus O-RADS categories. The diagnostic performance of senior radiologists was not included in the exploratory analysis of benign versus malignant discrimination, acknowledging the information imbalance between the models (text-only) and the radiologists (images + text).

### 2.8 Statistical analysis

Statistical analyses were primarily performed using SPSS (version 27.0; IBM Corp., Armonk, NY, USA). Prior to analysis, the three repeated outputs from each model per report were aggregated by majority voting to obtain a single output per mass. For structured report generation and O-RADS classification, the unit of analysis was each individual mass (n = 295); for benign/malignant discrimination, the unit was each patient (n = 215) to avoid clustering. Inter−model agreement for ordinal outcomes (structured report grades, O−RADS categories, and management recommendations) was assessed using weighted Cohen‘s kappa with quadratic weighting. Paired comparisons between the two models were analyzed using McNemar‘s test. Diagnostic performance was assessed by receiver operating characteristic analysis with area under the curve (AUC) calculations, with 95% confidence intervals directly obtained from SPSS output. Pairwise comparisons of AUCs were performed using DeLong’s nonparametric test implemented in R (version 4.3.1) with the pROC package, as SPSS does not natively support this test. For secondary outcomes (O-RADS accuracy and management recommendations), a Benjamini−Hochberg correction was applied (significance threshold P < 0.017). The primary outcome (structured report generation) was assessed at P < 0.05. For the stability analysis of the three repeated runs (**Supplementary Table S1**), weighted Fleiss’ Kappa with quadratic weighting was used to assess inter−run reproducibility, as recommended for ordinal outcomes, with analyses performed in R (version 4.3.1). Unclassifiable outputs were counted as incorrect in the accuracy calculation (**Table 1**). All tests were two-sided.

## 3 Results

### 3.1 Baseline characteristics

A total of 215 female patients (mean age 45 ± 14 years) with 295 AMs were enrolled in this study. The baseline characteristics of the study cohort are summarized in **Table 2**. Of the 295 masses, 184 (62.4%) were benign and 111 (37.6%) were malignant. The distribution of O−RADS categories by expert consensus and detailed histopathological subtypes are presented in **Table 2**.

**Table 2:** Baseline characteristics of the study cohort.

Category	Characteristic	Value
Patients	Total patients, n	215
	Age, mean ± SD (range), years	45 ± 14 (18–84)
	Menopausal status, n (%)	
	Premenopausal	136 (63.3%)
	Postmenopausal	79(36.7%)
Mass multiplicity, n(%)	Single mass	160(74.4%)
	Multiple masses (≥2)	55 (25.6%)
Mass Laterality, n (%)	Unilateral	168(78.1%)
	Bilateral	47(21.9%)
Masses	Total masses, n	295
O-RADS category distribution, n (%)	O-RADS 1	0(0%)*
	O-RADS 2	79(26.8%)
	O-RADS 3	84(28.5%)
	O-RADS 4	83(28.1%)
	O-RADS 5	49(16.6%)
Histopathological subtype, n (%)	Benign	184 (62.4%)
	Endometriotic cyst	55(29.9%)
	Mature cystic teratoma	33(17.9%)
	Serous cystadenoma	24(13.0%)
	Mesosalpingeal cyst	19(10.3%)
	Mucinous cystadenoma	10(5.4%)
	Fibroma/Thecoma	8(4.3%)
	Paramesonephric cyst	7(3.8%)
	Corpus luteum cyst	3 (1.6%)
	Corpus luteum hematoma	3 (1.6%)
	Follicular cyst	3 (1.6%)
	Luteinized cyst	2 (1.1%)
	Other benign lesions	17 (9.2%)
	Malignant	87(29.5%)
	High-grade serous carcinoma	28(32.2%)
	Low-grade serous carcinoma	10(11.5%)
	Mucinous carcinoma	9(10.3%)
	Metastatic malignant tumor	9(10.3%)
	Malignant sex cord-stromal tumors	7(8.0%)
	Endometrioid carcinoma	5 (5.7%)
	Clear cell carcinoma	3(3.4%)
	Malignant tumor of the fallopian tube	2(2.3%)
	Malignant germ cell tumors	2(2.3%)
	Other malignant lesions	12(13.8%)
	Borderline tumors, n (%)	24(8.1%)†

*O-RADS 1 cases (normal ovaries/simple follicles) were not included, as only surgically confirmed lesions were eligible. †Borderline tumors were classified as malignant for the benign versus malignant discrimination analysis. Percentages may not sum to 100% due to rounding.

### 3.2 Model performance: structured report generation (primary outcome)

The performance of the two models in generating structured ultrasound reports was evaluated at the report level (n = 215) and is shown in **Table 1**. GPT−4 showed numerically better performance than DeepSeek−R1 in generating complete structured reports (99.5% vs. 96.7%), though the difference did not reach statistical significance (P = 0.070). Both models demonstrated high completeness rates, with GPT−4 achieving 99.5% (214/215) and DeepSeek−R1 achieving 96.7% (208/215). The detailed distribution of structured report grades is presented in **Table 1**. The distribution of structured report grades across the three repeated runs is presented in **Supplementary Table S2**.

### 3.3 Model performance: O-RADS classification and management recommendations (secondary outcomes)

For O−RADS classification and management recommendations, performance was evaluated at the mass level (n = 295), with results summarized in **Table 1**. For O−RADS classification, DeepSeek−R1 outperformed GPT−4 (64.4% vs. 52.9%, P < 0.001). The confusion matrices for O−RADS classification by DeepSeek−R1 and GPT−4 against expert consensus are presented in **Table 3a** and **Table 3b**, respectively. DeepSeek−R1 showed notably better performance in O−RADS 4 categories, with a higher correct classification rate compared to GPT−4. Unclassifiable outputs were observed in 2/295 cases for DeepSeek−R1 and 12/295 cases for GPT−4, and were counted as incorrect in the accuracy calculation (**Table 1**).

**Table 3A:** Confusion matrix for O-RADS classification: DeepSeek-R1 vs. expert consensus.

DeepSeek-R1	Expert consensus			
	O-RADS 2	O-RADS 3	O-RADS 4	O-RADS 5
O-RADS 2	38(48.1%)	10(11.9%)	1(1.2%)	1(2.0%)
O-RADS 3	26(32.9%)	42(50%)	4(4.8%)	0(0%)
O-RADS 4	14(17.7%)	32(38.1%)	69(83.1%)	16(32.7%)
O-RADS 5	0(0%)	0(0%)	9(10.8%)	31(63.3%)

Valid O-RADS outputs were obtained for 293 of 295 masses (2 unclassifiable, 2/295). Column percentages are shown. O-RADS 1 not included. Percentages may not sum to 100% due to rounding.

**Table 3B:** Confusion matrix for O-RADS classification:GPT-4 vs.expert consensus.

GPT-4	Expert consensus			
	O-RADS 2	O-RADS 3	O-RADS 4	O-RADS 5
O-RADS 2	23(29.1%)	12(14.3%)	1(1.2%)	0(0%)
O-RADS 3	23(29.1%)	26(31.0%)	4(4.8%)	0(0%)
O-RADS 4	25(31.6%)	45(54.2%)	55(66.3%)	6(12.2%)
O-RADS 5	0(0%)	0(0%)	21(25.3%)	42(85.7%)

Valid O-RADS outputs were obtained for 283 of 295 masses (12 unclassifiable, 12/295). Column percentages are shown. O-RADS 1 not included. Percentages may not sum to 100% due to rounding.

For management recommendations, DeepSeek−R1 also demonstrated superior performance compared to GPT−4 (66.4% vs. 58.0%, P = 0.007; **Table 1**). The appropriateness of management recommendations was evaluated against O−RADS guidelines (2022 version), with DeepSeek−R1 providing more consistently appropriate recommendations than GPT−4. The distribution of outputs across the three repeated runs for O‑RADS classification and management recommendations is presented in **Supplementary Table S2**.

### 3.4 Stability analysis of three repeated runs

The reproducibility of the three repeated runs was assessed as a secondary analysis and is reported in **Supplementary Table S1**. The Complete Agreement rates (three runs identical) were 81.4% and 84.2% for DeepSeek-R1 and GPT-4, respectively, on structured reports, and exceeded 60% for both models across all endpoints. The modal agreement rates (defined as at least two of three runs agreeing) exceeded 90% for both models across all endpoints, respectively, indicating excellent practical reproducibility. Of note, the weighted Fleiss’Kappa values for structured reports were low (0.138 for DeepSeek-R1 and −0.005 for GPT-4), which is attributable to the highly skewed distribution of the outcome—the vast majority of outputs were consistently classified as "Structured". Under such near-constant marginal distributions, Kappa is statistically conservative and may approach or fall below zero, even when raw agreement rates are high. Therefore, the Complete Agreement and Modal Agreement rates provide a more clinically meaningful assessment of reproducibility for this endpoint. The weighted Fleiss‘Kappa values for O-RADS and management recommendations are also provided in **Supplementary Table S1** for reference.

### 3.5 Error analysis

The error analysis stratified by O−RADS category, pathological type, and mass multiplicity is presented in **Supplementary Table S4**. For single−mass reports, the error rates were 32.5% for DeepSeek−R1 and 41.3% for GPT−4. For reports containing multiple masses, the error rates increased to 60.0% for DeepSeek−R1 and 78.2% for GPT−4. Across both report types, DeepSeek−R1 demonstrated a lower error rate than GPT−4, with the performance gap being more pronounced in multiple−mass reports (18.2% absolute difference) compared to single−mass reports (8.8% absolute difference). This finding suggests that DeepSeek−R1 is more robust to report complexity.

### 3.6 Benign versus malignant discrimination (exploratory analysis)

**Figure 2:**
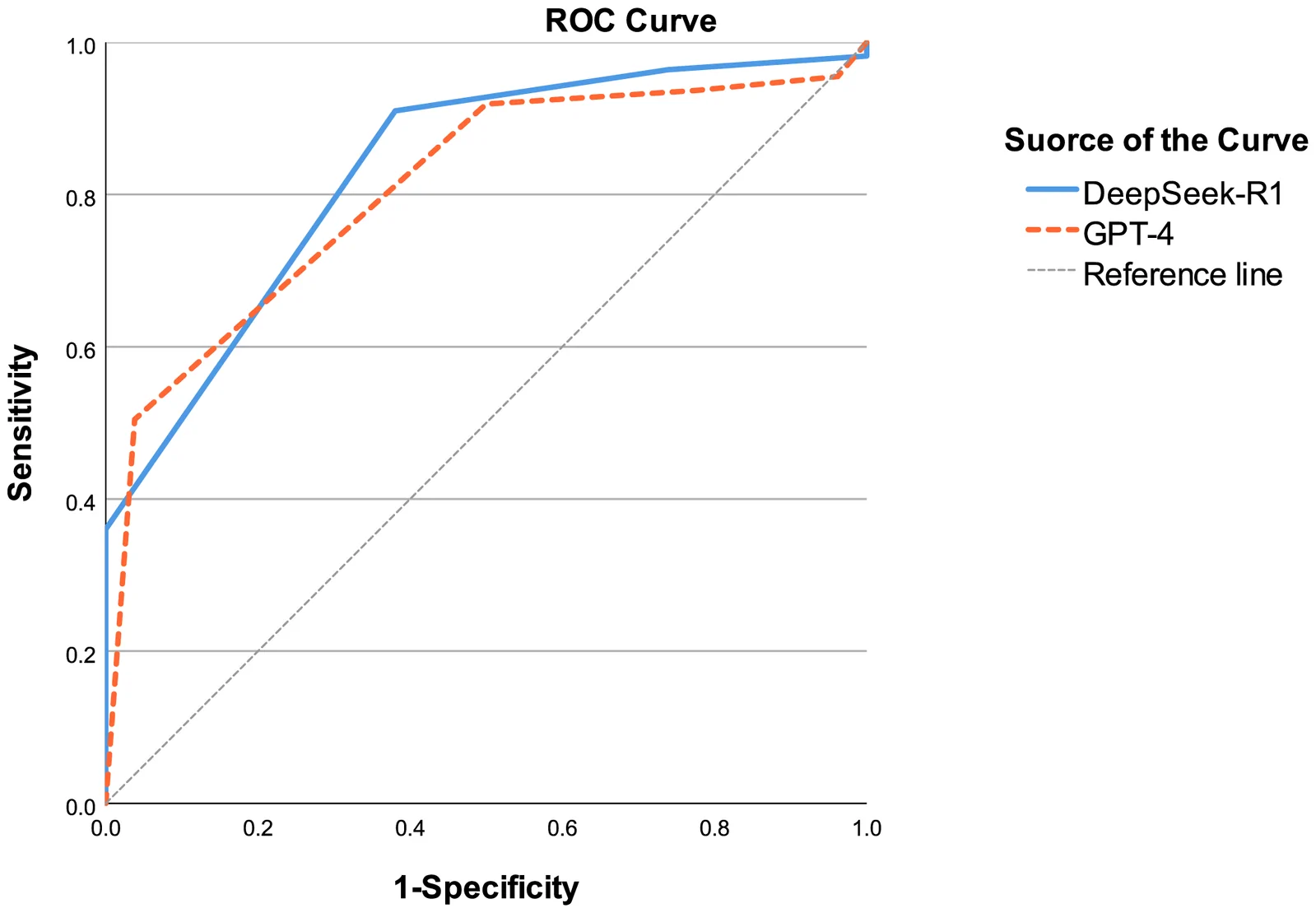
ROC analysis of DeepSeek-R1 and GPT-4 for benign-malignant differentiation of adnexal masses.

The ROC analysis for benign versus malignant discrimination was performed at the patient level (n = 215) and is shown in **Figure 2** and **Table 4**. DeepSeek−R1 demonstrated good discrimination ability with an AUC of 0.897 (95% CI: 0.863–0.931), while GPT−4 achieved an AUC of 0.825 (95% CI: 0.776–0.873). The difference between the two models was statistically significant (P = 0.040). Both models demonstrated AUC values > 0.80, indicating good overall discrimination ability for benign versus malignant classification. The sensitivity, specificity, positive predictive value, negative predictive value, and accuracy at the optimal Youden cut−offs are summarized in **Table 4**.

**Table 4:** Comparison of the ability to predict benign and malignant characteristics among DeepSeek-R1 and GPT-4.

*Variable*	DeepSeek-R1	GPT-4	P value*
AUC (95% CI)	0.897 (0.863–0.931)	0.825 (0.776–0.873)	0.040
Sensitivity (%)	95.5%	48.6%	
Specificity (%)	67.9%	96.7%	
PPV (%)	64.2%	90.0%	
NPV (%)	96.2%	75.7%	
Accuracy (%)	78.3%	78.6%	

*P < 0.05 was considered significant.

## 4 Discussion

This study confirms that AI systems based on LLMs demonstrate promising potential in generating structured reports for AMs, automating O-RADS categories, and providing clinical management recommendations. These systems can efficiently transform unstructured or semi-structured free-text input into standardized output using simple instructions. However, the observed performance gaps across different tasks highlight the current limitations of text-only LLM-based classification and underscore the need for further refinement before clinical implementation. The workflow efficiency and workload reduction benefits suggested by our findings should be interpreted with caution, as these outcomes were not directly evaluated in the present study.

The value of structured reporting in medical imaging has been widely recognized. Its advantages lie in enhancing the standardization, comparability, and data mining potential of diagnostic reports, ultimately aiming to optimize the efficiency of clinical decision support, report quality control management, research data integration, and interdisciplinary communication (23–27). However, its wide adoption and effective implementation in routine clinical practice have encountered significant resistance due to various factors, and it has even, to some extent, increased the workload of reporting practitioners. LLMs with their capabilities in natural language understanding and generation, offer potential for automating report generation or assisting in the construction of structured reports (28–30). Recent studies have demonstrated the feasibility of AI-based risk stratification for adnexal lesions using conventional models and LLMs (12–14). Building on this foundation, we investigated the feasibility of using DeepSeek**-**R1 and GPT**-**4 to generate structured ultrasound reports from unstructured free**-**text ultrasound reports. Our results demonstrated that GPT**-**4 showed numerically better performance than DeepSeek**-**R1 in generating structured reports, though the difference did not reach statistical significance (P = 0.070). However, DeepSeek**-**R1 outperformed GPT-4 in O-RADS classification accuracy and management recommendations.

Regarding the stability and performance in generating structured ultrasound reports, no statistically significant difference was observed between DeepSeek-R1 and GPT-4. The Complete Agreement rates (three runs identical) exceeded 80% for both models, and the Modal Agreement rates (at least two of three runs agreeing) exceeded 95%, indicating excellent practical reproducibility. Although the weighted Fleiss‘ Kappa values for structured reports were low due to the highly skewed distribution of the outcome (the vast majority of outputs were consistently classified as ‘Structured’), the raw agreement rates provide a more clinically meaningful assessment of reproducibility. Both models demonstrated notably high capability and stability in producing structured reports.

The study results revealed that both DeepSeek-R1 and GPT-4 demonstrated strong consistency in their O-RADS category assignments. However, regarding accuracy, although DeepSeek-R1 outperformed GPT-4, the overall accuracy of both models (64.4% for DeepSeek-R1 and 52.9% for GPT-4) suggests that text-only classification remains challenging. This is likely due to the fact that O-RADS categories integrate complex morphological, hemodynamic, and clinical contextual knowledge, whereas these two models could only rely on the free-text descriptions of AMs for judgment. The error analysis (**Supplementary Table S4**) revealed that both models exhibited substantially higher error rates in multiple-mass reports compared to single-mass reports (DeepSeek-R1: 32.5% to 60.0%; GPT-4: 41.3% to 78.2%). This pattern suggests that the presence of multiple masses within a single report poses a significant challenge for text-based O-RADS classification, with a more pronounced impact on GPT-4. The confusion matrices (**Tables 3a**, **b**) further illustrate the distinct error patterns of the two models.

Previous studies have proposed that AI-generated well-structured reports with appropriate professional terminology and clear communication are essential components of effective medical reporting. This capability is particularly crucial for enhancing patient comprehension and enabling informed decision-making aligned with their preferences (31, 32). Regarding the consistency and accuracy of management recommendations, both DeepSeek-R1 and GPT-4 demonstrated substantial potential in output agreement, with GPT-4 exhibiting particularly notable performance. However, the accuracy of management recommendations (66.4% for DeepSeek-R1 and 58.0% for GPT-4) was suboptimal, likely because both models provided management recommendations based solely on O-RADS categories. Therefore, the clinical soundness of management recommendations delivered by both models is intimately associated with the diagnostic accuracy of O-RADS classifications. This interdependence highlights a critical limitation of text-only LLM-based systems: their performance is constrained by the accuracy of intermediate classification tasks.

Regarding benign versus malignant discrimination, both models demonstrated good discrimination ability with AUC values > 0.80 (DeepSeek-R1: 0.897; GPT-4: 0.825). The difference between the two models was statistically significant (P = 0.040), suggesting that DeepSeek-R1 may be more sensitive in detecting malignancy from text-only reports. However, these results should be interpreted with caution, as the models were evaluated on text-based reports without access to original ultrasound images. This limitation is particularly relevant given that O-RADS classification in clinical practice relies heavily on imaging features that may not be fully captured in narrative reports.

It is important to acknowledge the information imbalance between the LLMs and the reference standard used in this study. The models processed only text-based reports, whereas the senior radiologists who established the reference standards for O-RADS classification and management recommendations had access to both original ultrasound images and free-text reports. This asymmetry likely favored the radiologists and should be considered when interpreting the comparative results. The radiologists‘ performance serves as a reference benchmark rather than a direct comparator under identical information conditions. Future studies integrating image-based features with text-based LLM processing are needed to more accurately assess the potential of these models in clinical settings.

The present investigation explores the initial efficacy and challenges of utilizing LLMs to assist in generating structured ultrasound reports. It aims to evaluate the feasibility and value of leveraging this technology to promote the clinical adoption of structured reporting. Concurrently, analysis revealing suboptimal performance in areas such as O-RADS categories accuracy and the provision of management recommendations provides valuable feedback for refining and optimizing the underlying algorithms. These improvements are essential for enhancing the accuracy and reliability of the generated reports. The findings of our study align with recent reports on LLM-based O-RADS classification (13) and AI-driven ultrasound detection of ovarian cancer (14), which have similarly highlighted the potential of AI in gynecologic imaging while emphasizing the need for careful validation and integration with image-based modalities.

## 5 Limitations

Several limitations should be acknowledged. First, this was a retrospective, single-center study, which may introduce selection bias and institution-specific reporting patterns, including spectrum bias due to inclusion of only surgically treated patients; external validation in multi-center cohorts is needed. Second, the models were evaluated on text reports only, without access to original ultrasound images, and the radiologists who established the reference standards had access to both images and text—this information imbalance should be considered when interpreting the comparative results. Third, LLM performance is sensitive to prompting strategies, parameter settings, and stochasticity; the prompt used was relatively simple and did not incorporate advanced techniques such as few-shot learning. Fourth, the reference standards for O-RADS and management recommendations were based on expert consensus rather than an independent gold standard, and some degree of subjective judgment was unavoidable. Fifth, the sample size was modest, and the model versions evaluated may become outdated rapidly; furthermore, this study did not assess clinical impact or cost-effectiveness, which require prospective investigation.

In conclusion, our study demonstrates that both DeepSeek-R1 and GPT-4 exhibit robust performance in generating structured ultrasound reports for AMs, with GPT-4 showing a numerical advantage that did not reach statistical significance. DeepSeek-R1 demonstrated superior accuracy in O-RADS classification and management recommendations compared to GPT-4. However, their capabilities in AMs risk assessment and providing corresponding clinical management recommendations require further optimization, particularly in complex reports containing multiple masses. The exploratory analysis of benign versus malignant discrimination showed promising discrimination ability (AUC > 0.80 for both models), though this was based on text-only inputs without image integration. Consequently, clinical application warrants caution and should incorporate expert oversight and validation in prospective, multi-center settings with integrated imaging data.

## Data Availability

The raw data supporting the conclusions of this article will be made available by the authors, without undue reservation.
