# Supplementary material for: Comparative assessment of DeepSeek-R1 and GPT-4 for structured ultrasound reporting of adnexal masses
Source: Front Oncol. 2026 Aug 11;16:1904488. doi: 10.3389/fonc.2026.1904488 (PMC13503586; doi:10.3389/fonc.2026.1904488)
Supplement: Supplementary file 1 [file DataSheet1.pdf]

Example of free-text to structured report conversion by DeepSeek-R1 for an adnexal mass.

<table>
<tr><td>FREE- TEXT REPORT</td><td colspan="2">A 57-year-old naturally postmenopausal female patient presented with abdominal distension, poor appetite, and postprandial discomfort for over two weeks, accompanied by afternoon low back pain and occasional abdominal pain. She had not received any prior treatment. Ultrasound examination revealed a normal-sized uterus. Both ovaries were not clearly visualized. In the left adnexal region, a cystic lesion measuring approximately 76×66 mm was noted, with ill-defined borders, irregular shape, and unclear demarcation from the uterus. Peripheral hypoechoic areas and several papillary projections were observed, the largest with a broad base measuring approximately 10 mm in width and 6 mm in height. Color Doppler flow signals were detected around the lesion (RI: 0.54). No free fluid was noted in the cul-de-sac.</td></tr>
<tr><td rowspan="5">STRUCTURED REPORT</td><td>PATIENT INFORMATION</td><td>Age: 57 years
Gender: Female
Menopausal status: Naturally postmenopausal</td></tr>
<tr><td>CLINICAL HISTORY</td><td>Presented with abdominal distension, poor appetite, and postprandial discomfort for over 2 weeks, with afternoon low back pain and occasional abdominal pain. No prior treatment.</td></tr>
<tr><td rowspan="3">ULTRASOUND FINDINGS</td><td>Uterus: Normal size.
Left adnexal region: a cystic lesion measuring approximately 76×66 mm, with ill- defined borders, irregular shape, and unclear demarcation from the uterus.
Right ovary: Not clearly visualized.</td></tr>
<tr><td>Mass characteristics :
Location: Left adnexal region
Size: 76×66 mm
Cystic components: Predominantly cystic with solid components
Inner wall: Irregular, with papillary projections
Solid component: Present (papillary projections; largest: 10 mm base × 6 mm height)
Papillary projections: Yes (multiple; largest: 10 mm base × 6 mm height)
Internal echoes: Present (low- level echoes with papillary projections)
Posterior acoustic features: Not specified (not described in the original report)
Color Doppler flow: Detected around the lesion (RI: 0.54)</td></tr>
<tr><td>Other findings :No free fluid in the cul- de- sac.</td></tr>
<tr><td>O- RADS CATEGORY</td><td colspan="2">O- RADS 4</td></tr>
<tr><td>MANAGEMENT RECOMMENDATION</td><td colspan="2">Biopsy or surgical consultation recommended for further evaluation due to the presence of solid components (papillary projections) and vascularity (RI: 0.54) suggestive of intermediate-high risk features.</td></tr>
</table>
