# Supplementary material for: Comparative assessment of DeepSeek-R1 and GPT-4 for structured ultrasound reporting of adnexal masses
Source: Front Oncol. 2026 Aug 11;16:1904488. doi: 10.3389/fonc.2026.1904488 (PMC13503586; doi:10.3389/fonc.2026.1904488)
Supplement: Supplementary file 2 [file Table1.docx]

Supplementary Material S1：Predefined structured report template for adnexal masses

This template was used as the reference standard for evaluating the completeness and accuracy of structured reports generated by DeepSeek‑R1 and GPT‑4. The template contains the following fields, which were derived from routine clinical reporting standards and the ACR O‑RADS guidelines (2022 version).

**Structured ultrasound report – adnexal masses**

| **Patient Information** | Age: [years]  Menopausal status: [premenopausal / postmenopausal] |
| --- | --- |
| **Clinical History** | Presenting symptoms:[e.g.,pelvic pain, bloating, asymptomatic]  Relevant medical history:[e.g., previous ovarian surgery, family history of ovarian cancer] |
| **Ultrasound Findings** | **Global Ultrasound Description:**  Uterus: [normal / abnormal findings]  Contralateral ovary: [normal / abnormal findings]  Other adnexal findings: [e.g., free fluid, peritoneal thickening] |
|  | **Mass Characteristics** (for each mass)  Location: [right / left / bilateral]  Size (mm): [largest diameter × second × third]  Shape: [oval / round / irregular]  Margins: [smooth / irregular / ill‑defined]  Internal echogenicity: [anechoic/hypoechoic/hyperechoic/mixed/cystic with solid component]  Posterior acoustic features: [enhancement/shadowing/no change/ combined]  Color Doppler Flow: [no flow / minimal flow / moderate flow / rich flow] |
| **O‑RADS Category** | O‑RADS Category 1/2/3/4/5 |
| **Management Recommendation** | routine follow‑up / short‑interval follow‑up / further imaging / biopsy / surgical referral] |
| **Impression** | [Summary of key findings and clinical significance] |

**Evaluation criteria used in the study:**

| Category | Definition |
| --- | --- |
| Structured and correct | All fields present and content matched the original report |
| Partially structured | Most but not all fields present; no erroneous information |
| Unstructured or erroneous | Lacked clear structure or contained clinically incorrect information |
