# Supplementary material for: Comparative assessment of DeepSeek-R1 and GPT-4 for structured ultrasound reporting of adnexal masses
Source: Front Oncol. 2026 Aug 11;16:1904488. doi: 10.3389/fonc.2026.1904488 (PMC13503586; doi:10.3389/fonc.2026.1904488)
Supplement: Supplementary file 3 [file Table2.docx]

**Complete prompt template**

1. **prompt：**

“Please generate a structured medical ultrasound report based on the following free-text description of an adnexal mass. The report should include patient information, clinical history,ultrasound findings, O-RADS categories, and management recommendations based on the O-RADS guidelines (2022 version).” Free‑text report: [insert report text here]

## 2.Model Specifications and Access Settings

Two large language models were evaluated: DeepSeek‑R1 and GPT‑4.

DeepSeek‑R1: version R1-202505 (DeepSeek Inc., Beijing, China). Accessed via the official API from June 1 to June 10, 2025.

GPT‑4: version GPT-4-2024-04-09 (OpenAI Inc., San Francisco, CA, USA). Accessed via the OpenAI API during the same period (June 1–10, 2025).

**3.Handling of Repeated Runs**

Each free‑text report was independently processed three times per model. For each model–report pair, majority voting was used to determine the final output (i.e., the result that appeared at least twice among the three runs; if all three outputs differed, the first run was used as the tie‑breaker).
