# Supplementary material for: Comparative assessment of DeepSeek-R1 and GPT-4 for structured ultrasound reporting of adnexal masses
Source: Front Oncol. 2026 Aug 11;16:1904488. doi: 10.3389/fonc.2026.1904488 (PMC13503586; doi:10.3389/fonc.2026.1904488)
Supplement: Supplementary file 4 [file Table3.docx]

Supplementary Table S1. Stability analysis of three repeated runs using weighted Fleiss**‘** Kappa

| Variable | Model | N | Complete Agreement (%) | Modal Agreement Rate | Mean pairwise agreement | Weighted Fleiss’ Kappa (95% CI)* |
| --- | --- | --- | --- | --- | --- | --- |
| Structured Report | DeepSeek‑R1 | 215 | 81.40% | 100% | 87.6% | 0.138（0.013, 0.273） |
|  | GPT‑4 | 215 | 84.20% | 100% | 89.5% | -0.005（-0.054, 0.075） |
| O‑RADS | DeepSeek‑R1 | 295 | 66.10% | 100% | 77.4% | 0.522（0.446, 0.594） |
| Categories | GPT‑4 | 295 | 70.80% | 98.6% | 80.1% | 0.631（0.565, 0.694） |
| Management | DeepSeek‑R1 | 295 | 61.00% | 96.6% | 72.9% | 0.524（0.449, 0.591） |
| Recommendations | GPT‑4 | 295 | 77.60% | 99.0% | 84.7% | 0.741（0.680, 0.796） |

*Complete agreement refers to the proportion of cases where all three runs produced identical outcomes. Weighted Fleiss‘Kappa with quadratic weighting was used for ordinal outcomes (structured report grades: structured/partially structured/unstructured; o-RADS categories:correct/incorrect/unclassifiable; management recommendations: reasonable/partially reasonable/unreasonable or not provided).

“The low weighted kappa values for structured reports are attributable to the highly skewed distribution of the outcome (the vast majority of three-run outputs were consistently classified as ‘Structured’), which renders kappa statistically conservative. The Modal Agreement Rate (≥2 of 3 runs agreeing) and Complete Agreement (3 of 3 runs identical) were≥80% across all endpoints, confirming excellent practical reproducibility.
