# Supplementary material for: Comparative assessment of DeepSeek-R1 and GPT-4 for structured ultrasound reporting of adnexal masses
Source: Front Oncol. 2026 Aug 11;16:1904488. doi: 10.3389/fonc.2026.1904488 (PMC13503586; doi:10.3389/fonc.2026.1904488)
Supplement: Supplementary file 5 [file Table4.docx]

Supplementary Table S2. Distribution of the Scale for Structured Reports, O-RADS Categories, Management Recommendations, and Predictive Ability of DeepSeek-R1 and GPT-4.

(a)Performance of DeepSeek-R1

| Variable | Scale | DeepSeek-R1 | | | |
| --- | --- | --- | --- | --- | --- |
|  |  | Run1 | Run2 | Run3 | Total |
| Structured Reports | Structured | 84.2%（181/215) | 89.3%(192/215) | 88.8%(191/215) | 87.5%(564/645) |
|  | Structured but incomplete | 3.3%(7/215) | 1.4%(3/215) | 1.4%(3/215) | 2.0%(13/645) |
|  | Unstructured or contains incorrect information | 12.5%(27/215) | 9.3%(20/215) | 9.8%(21/215) | 10.5%(68/645) |
| O-RADS Categories | Correct | 66.8%(197/295) | 60.7%(179/295) | 61.0%(180/295) | 62.8%(556/885) |
|  | Incorrect | 32.9%(97/295) | 38.6%(114/295) | 38.0%(112/295) | 36.5%(323/885) |
|  | Not categorized | 0.3%(1/295) | 0.7%(2/295) | 1.0%(3/295) | 0.7%(6/885) |
| Management  Recommendations | Reasonable | 65.4%(193/295) | 61.0%(180/295) | 65.4%(193/295) | 64.0%(566/885) |
|  | Partially reasonable | 13.2%(39/295) | 13.2%(39/295) | 5.8%(17/295) | 10.7%(95/885) |
|  | Unreasonable or not provided | 21.4%(63/295) | 25.8%(76/295) | 28.8%(85/295) | 25.3%(224/885) |

(b)Performance of GPT-4

| Variable | Scale | GPT-4 | | | |
| --- | --- | --- | --- | --- | --- |
|  |  | Run1 | Run2 | Run3 | Total |
| Structured Reports | Structured | 90.7%(195/215) | 89.3%(192/215) | 90.2%(194/215) | 90.1%(581/645) |
|  | Structured but incomplete | 6.0%(13/215) | 7.4%(16/215) | 5.1%(11/215) | 6.2%(40/645) |
|  | Unstructured or contains incorrect information | 3.3%(7/215) | 3.3%(7/215) | 4.7%(10/215) | 3.7%(24/645) |
| O-RADS Categories | Correct | 53.9%(159/295) | 52.9%(156/295) | 51.5%(152/295) | 52.8%(467/885) |
|  | Incorrect | 42.0%(124/295) | 42.7%(126/295) | 42.7%(126/295) | 42.5%(376/885) |
|  | Not categorized | 4.1%(12/295) | 4.4%(13/295) | 5.8%(17/295) | 4.7%(42/885) |
| Management  Recommendations | Reasonable | 59.3%(175/295) | 56.6%(167/295) | 59.3%(175/295) | 58.4%(517/885) |
|  | Partially reasonable | 8.8%(26/295) | 10.2%(30/295) | 11.9%(35/295) | 10.3%(91/885) |
|  | Unreasonable or not provided | 31.9%(94/295) | 33.2%(98/295) | 28.8%(85/295) | 31.3%(277/885) |
