# Supplementary material for: Comparative assessment of DeepSeek-R1 and GPT-4 for structured ultrasound reporting of adnexal masses
Source: Front Oncol. 2026 Aug 11;16:1904488. doi: 10.3389/fonc.2026.1904488 (PMC13503586; doi:10.3389/fonc.2026.1904488)
Supplement: Supplementary file 6 [file Table5.docx]

**Supplementary Table S3.** Model performance stratified by report complexity

| **Condition** | **N (Reports)** | **DeepSeek‑R1 Accuracy (%)** | **GPT‑4 Accuracy (%)** | **P value*** |
| --- | --- | --- | --- | --- |
| Single mass | 160 | 108（67.5%） | 94（58.8%） | 0.034 |
| Multiple masses, same O‑RADS | 23 | 12（52.2%） | 10（43.5%） | 0.687 |
| Multiple masses, different O‑RADS | 32 | 10（31.3%） | 2（6.3%） | 0.008 |

*For each complexity stratum, pairwise comparisons between the two models were performed using McNemar ’s test. Given the exploratory nature of the stratified analysis, the Benjamini‑Hochberg procedure was applied to control the false discovery rate for the three comparisons, with a significance threshold of P < 0.017.
