# Supplementary material for: Comparative assessment of DeepSeek-R1 and GPT-4 for structured ultrasound reporting of adnexal masses
Source: Front Oncol. 2026 Aug 11;16:1904488. doi: 10.3389/fonc.2026.1904488 (PMC13503586; doi:10.3389/fonc.2026.1904488)
Supplement: Supplementary file 7 [file Table6.docx]

**Supplementary Table S4. Error analysis of model O‑RADS classification**

| Stratification | Variable | N | DeepSeek‑R1 Error Rate (%) | GPT‑4 Error Rate (%) | P value |
| --- | --- | --- | --- | --- | --- |
| O‑RADS category | O‑RADS 2 | 79 | 42(53.2%) | 58(73.4%) | 0.002 |
|  | O‑RADS 3 | 84 | 40(47.6%) | 54(64.3%) | 0.07 |
|  | O‑RADS 4 | 83 | 7(8.4%) | 22(26.5%) | 0.01 |
|  | O‑RADS 5 | 49 | 16(32.7%) | 5(10.2%) | 0.03 |
| pathological type | Benign | 184 | 70(38.0%) | 99(53,8%) | ＜0.001 |
|  | Borderline | 24 | 5(20.8%) | 7(29.2%) | 0.5 |
|  | Malignant | 87 | 5(5.7%) | 2(2.3%) | 0.25 |
| mass multiplicity | Single mass | 160 | 52(32.5%) | 66(41.3%) | 0.034 |
|  | Multiple masses | 55 | 33(60%) | 43(78.2%) | 0.013 |
